# *Trichinella papuae* in Saltwater Crocodiles (*Crocodylus porosus*) of Papua New Guinea

**DOI:** 10.3201/eid1008.040082

**Published:** 2004-08

**Authors:** Edoardo Pozio, Ifor L. Owen, Gianluca Marucci, Giuseppe La Rosa

**Affiliations:** * Istituto Superiore di Sanità, Rome, Italy;; † National Agriculture Quarantine and Inspection Authority, Port Moresby, Papua New Guinea

**Keywords:** letter, *Trichinella papuae*, saltwater crocodiles, *Crocodylus porosus*, reptiles, wild pig, meat, Papua New Guinea, epidemiology, PCR

**To the Editor:** Until 1995, reptiles were not known to be hosts of *Trichinella*; however, in that year *Trichinella* was detected in 40% of farm-raised crocodiles (*Crocodylus niloticus*) in Zimbabwe. These crocodiles were infected with a new species, *T. zimbabwensis*, which was experimentally infective in mammals, including primates (1).

The infection of reptiles with *Trichinella* species that are potentially infective for humans has become more important since demand for the meat of crocodiles, caimans, and alligators has increased in many areas of the world. This trend has resulted in the development of national breeding programs in more than 30 countries in North, Central, and South America; Africa; Asia; and Australia (2), which generated an income of approximately $60 million in 1998 (3).

In 1999 in Papua New Guinea, wild and domestic pigs infected with a new species, *T. papuae*, were found (4,5); this new species was capable of completing its life cycle in reptiles that were infected experimentally (6). *Trichinella* infection has also been found in farm-raised saltwater crocodiles (*C. porosus*) in Papua New Guinea, where a national program for crocodile meat and skin products exists.

Papua New Guinea has one crocodile breeding farm that processes approximately 6,000 animals per year. Following the discovery of *Trichinella-*infected crocodiles in Zimbabwe, the Australian government requested that Papua New Guinea conduct *Trichinella* testing on the crocodile meat exported to Australia. Muscle samples from crocodiles were digested by pepsin and HCl solution according to the standard technique (7). When available, approximately 100 larvae from each infected crocodile were given by mouth to laboratory rats, and 10–20 larvae were stored in 90% ethyl alcohol for molecular identification. Multiplex polymerase chain reaction (PCR) was used to characterize the larvae, according to a published protocol (8). The primer set oTsr1 and oTsr4 was used to amplify the expansion segment V of the large subunit ribosomal RNA (9). The larvae of all *Trichinella* reference strains were used as controls. PCR products were gel-purified and directly sequenced by using the same primers as those used for PCR amplification. All sequences were aligned by using the Clustal W program from OMIGA 2.0 (Accelrys, San Diego, CA). Final alignment of the expansion segment V sequences was performed manually so microsatellites could be compared.

Muscle samples from 118 saltwater crocodiles (46 farm-born, 71 wild-born and farm-raised, and 1 killed in the wild near the Bensbach River) were tested. All samples from the farm-born crocodiles were negative for *Trichinella*. Of the samples from the 72 wild-born crocodiles (including the 1 killed in the wild), 16 (22.2%) were positive for *Trichinella* larvae, with an average of 7 larvae/g in the biceps. All of the infected crocodiles originated in the Kikori area (Figure). The prevalence of *Trichinella* infection in crocodiles from this area was 32.0% (16/50). Samples from the remaining 21 wild-born and farm-raised crocodiles, and the 1 killed in the wild, were negative for *Trichinella*. These crocodiles originated in nine different locations (Figure).

**Figure:**
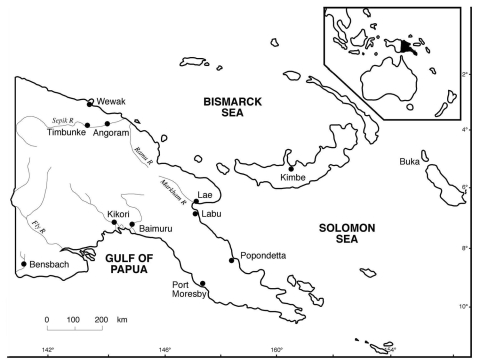
Papua New Guinea showing the areas of origin of the 72 wild-born saltwater crocodiles (*Crocodylus porosus*): 1 each from Baimuru, Angoram, Timbunke, Kimbe, Bensbach River, and Buka; 2 from Labu; 7 from Wewak; 7 from Popondetta; and 50 from Kikori.

PCR analysis showed that the parasites belonged to *T. papuae*. However, the crocodile isolates differed from the reference strain of this species by the deletion of a TG dinucleotide and by a single base mutation (G vs. A) in the expansion segment V sequence. Testing for *Trichinella* in crocodile meat has been conducted in Zimbabwe and Papua New Guinea only, and infected crocodiles have been found in both countries. Crocodiles in other parts of the world are also likely to be infected. Since both *T. zimbabwensis* and *T. papuae* infection can develop in reptiles and mammals, eating crocodile meat is a risk. In one region of Papua New Guinea, a high percentage of the local human population had anti-*Trichinella* antibodies (10). Moreover, the risk for human infection may be rising, given the increased marketing of meat from crocodiles, caimans, and alligators in many parts of the world (2). The meat of other carnivorous reptiles, although consumed in very few areas, may also represent a source of infection, as suggested by the large number of larvae of both *T. papuae* and *T. zimbabwensis* in the muscles of experimentally infected monitor lizards (6).

The presence of a TG dinucleotide in the expansion segment V sequence could be a useful marker for tracing the region of origin of infected meat. The infected crocodiles, all of which were born in the wild, likely acquired infection before they arrived on the farm, since none of the farm-born crocodiles was infected. In Zimbabwe, the source of infection was the *Trichinella*-infected crocodile meat that had been fed to the other crocodiles; the farm in Papua New Guinea does not engage in this practice, which would explain why none of its farm-born animals was infected.

This study shows the importance of implementing measures to prevent the spread of *Trichinella* infection. For instance, since both *T. papuae* and *T. zimbabwensis* can be easily transmitted from crocodiles to mammals, the discarded parts of crocodiles should be properly destroyed to avoid transmission to synanthropic animals, and the waste products should not be fed to domestic animals, unless the products are frozen or cooked before use. Crocodile-breeding farms should adopt the artificial digestion method used in many countries to screen pigs for *Trichinella* infection (7). Freezing crocodile meat, as practiced in Papua New Guinea, can also prevent infection because freezing destroys *T. papuae* and *T. zimbabwensis* larvae in muscles (1,4). By contrast, salting, drying, smoking, or preserving crocodile meat in brine will not destroy trichinellae; these curing methods are not standardized, and the survival of *Trichinella* larvae can depend on factors such as salt concentration, moisture, and temperature (7). Similarly, crocodile meat is frequently vacuum sealed, and the *Trichinella* larvae can retain their infectivity for several months in this environment (7).
